# Impact of ASA score misclassification on NSQIP predicted mortality: a retrospective analysis

**DOI:** 10.1186/s13741-017-0076-1

**Published:** 2017-12-11

**Authors:** Alex Helkin, Sumeet V. Jain, Angelika Gruessner, Maureen Fleming, Leslie Kohman, Michael Costanza, Robert N. Cooney

**Affiliations:** Department of Surgery, SUNY Upstate Medical University, 750 East Adams Street, Syracuse, NY 13206 USA

**Keywords:** ASA, NSQIP, Predicted mortality

## Abstract

**Background:**

The ASA physical classification score has a major impact on the observed/expected (O/E) mortality ratio in the NSQIP General Vascular Mortality Model. The difference in predicted mortality is greatest between ASAs 3 and 4. We hypothesized under-classified ASA scores significantly affect the O/E mortality.

**Methods:**

We conducted a retrospective review of NSQIP essential surgery cases from January 2014 to December 2014 (*n* = 1264) with mortality sub-analysis (*n* = 33) at our institution. We recorded transfer and emergency status and independently calculated the ASA score for mortalities using published definitions. A random sample of 50 survivors and 10 emergency survivors were reviewed and ASA recalculated. We performed statistical modeling to simulate the effects of ASA misclassifications. Statistical analysis was performed using JMP 10 and SAS 9.4.

**Results:**

ASA was under-classified in 18.2% of mortalities, most commonly ASAs 3 and 4. Sixteen percent of ASA 3 survivors were misclassified, including 60% in the emergency subgroup (*p* < 0.05 vs. elective cases). Patients transferred from other institutions were more likely to be emergency cases than non-transferred patients (43.5 vs. 7.84%, *p* < 0.05). Transferred patients had a higher proportion of ASAs 3–5 vs. ASAs 1–2 compared with non-transfers (84.38 vs. 49.76%, *p* < 0.05) Simulation data showed ASA misclassification underestimated predicted mortality by 2.5 deaths on average.

**Conclusion:**

ASA misclassification significantly impacts O/E mortality. With accurate ASA classification, observed mortality would not have exceeded expected mortality in our institution. Education regarding the impact of ASA scoring is critical to ensure accurate O/E mortality data at hospitals using NSQIP to assess surgical quality.

## Background

A current focus of health care systems is to improve the cost and quality of patient care. The American College of Surgeons National Surgical Quality Improvement Program (NSQIP®) is commonly used to collect and report data on institution-specific, risk-adjusted surgical outcomes. A systematic sampling approach is used to determine which surgical cases are selected for abstraction (Shiloach et al. 2010) based on the hospital’s specific program: Essentials, Procedure-Targeted, Small & Rural, or Pediatric. Participating institutions are provided with quarterly reports on risk-adjusted complication rates for a variety of postoperative occurrences including surgical site infections, renal failure, thromboembolic complications, cardiac events, readmission, and observed to expected (O/E) mortality. Institutional performance for specific complications are compared with other hospitals and assigned a decile rank. The ranking is reported as “Needs Improvement,” “As Expected,” or “Exemplary,” when compared with expected complication rates using standardized models, such as the NSQIP General Vascular Mortality Model (GVMM) for O/E mortality. Institutions can then use the NSQIP’s institution-specific benchmarking data to focus their quality improvement initiatives. NSQIP participation is effective in helping institutions identify potential problems in surgical care (Steinberg et al. 2008; Fink et al. 2002); however, in most instances, additional analyses by the participating hospitals are required to develop a better understanding of how best to prevent or decrease complications.(Schilling et al. 2008).

In 2014, our institution received a NSQIP report indicating a higher than expected observed-to-expected 30-day mortality rate and was subsequently assigned a “Needs Improvement” status. We routinely review all major surgical complications and mortalities through our surgical Morbidity and Mortality conference and were surprised to learn we ranked in the lowest decile for this category. Focusing on mortality, we first reviewed the surgical literature to aid in identifying factors that might impact predicted mortality (Fink et al. 2002) and then did chart reviews of all surgical mortalities in 2014. We initially abstracted data to get a better understanding of patient-specific risk factors and process-of-care variables that might affect mortality including transfer status, need for emergency surgery, use and timing of “do not resuscitate” (DNR) status, “procedure risk” (low, medium, or high), NSQIP and University Hospital Consortium predicted mortality, and finally the American Society of Anesthesia (ASA) physical status classification system.

Based on our initial review, we turned our focus to the ASA score (Table 1) (Durham et al. 2006) as a potential contributor to our higher than expected O/E mortality rate. The ASA score is assigned by the anesthesia team and provides a baseline metric for the fitness of a patient prior to undergoing surgery. The ASA score is an important predictor of mortality in surgical patients (Davenport et al. 2006; Davenport et al. 2005) and has been specifically validated for use in the NSQIP GVMM. The NSQIP rules for data entry require the SCR to use the ASA score recorded by the anesthesia team but allow the SCR to add the suffix E in cases where the surgical team documents the emergent nature of the surgical procedure, if not already documented in the “anesthesia assigned” ASA score. During our chart review of the 2014 mortalities, we identified several misclassified ASA scores, which greatly altered predicted mortality according to the NSQIP online preoperative risk calculator. Based on this finding, we hypothesized misclassified ASA scores falsely decreased the expected mortality and contributed to the increased 2014 NSQIP O/E mortality at our institution.

**Table 1:** American Society of Anesthesiologists physical classification system (Durham et al. 2006)

ASA physical status classification	Definition	Examples, including, but not limited to
ASA I	A normal healthy patient	Healthy, non-smoking, no or minimal alcohol use
ASA II	A patient with mild systemic disease	Mild diseases only without substantive functional limitations. Examples include (but not limited to) current smoker, social alcohol drinker, pregnancy, obesity (30 < BMI < 40), well-controlled DM/HTN, and mild lung disease
ASA III	A patient with severe systemic disease	Substantive functional limitations: one or more moderate to severe diseases. Examples include (but not limited to) poorly controlled DM or HTN, COPD, morbid obesity (BMI ≥ 40), active hepatitis, alcohol dependence or abuse, implanted pacemaker, moderate reduction of ejection fraction, ESRD undergoing regularly scheduled dialysis, premature infant PCA < 60 weeks, and history (> 3 months) of MI, CVA, TIA, or CAD/stents
ASA IV	A patient with severe systemic disease that is a constant threat to life	Examples include (but not limited to) recent (< 3 months) MI, CVA, TIA, or CAD/stents, ongoing cardiac ischemia or severe valve dysfunction, severe reduction of ejection fraction, sepsis, DIC, and ARD or ESRD not undergoing regularly scheduled dialysis
ASA V	A moribund patient who is not expected to survive without the operation	Examples include (but not limited to) ruptured abdominal/thoracic aneurysm, massive trauma, intracranial bleeding with mass effect, and ischemic bowel in the face of significant cardiac pathology or multiple organ/system dysfunction
ASA VI	A declared brain-dead patient whose organs are being removed for donor purposes	

## Methods

The study was approved by our institutional review board for exemption from review because it used retrospective, de-identified data. At our institution, 1264 general and vascular surgical cases were reported to NSQIP in 2014, which included 33 mortalities. The medical records of these 33 patients were reviewed by two surgery residents (AH and SJ) independently, who did not participate in any of the cases. ASA score was independently calculated by each reviewer and then discussed together to reach consensus based on published guidelines on the ASA website (asahq.org). In addition to ASA score, the following data were abstracted: transfer from another institution, the need for emergency surgery, DNR status and timing relative to death, and procedure risk. To determine factors significantly affecting predicted mortality, all patient factors that are part of the NSQIP online calculator were reviewed including procedure, age, gender, functional status, emergency status, wound class, steroid use, ascites present within 30 days of surgery, systemic sepsis present within 30 days of surgery, diabetes, hypertension, previous cardiac events, congestive heart failure, dyspnea on presentation, tobacco use, history of COPD, dialysis, acute renal failure, ventilator dependence, disseminated cancer, body mass index, and reclassification of the ASA score using the American Society of Anesthesiologists published guidelines (Table 1) (Durham et al. 2006). This analysis determined ASA was the major factor in NSQIP modeling, and discrepancies in classification lead to substantially different outcomes, specifically changes from ASA 3 to ASA 4. Changes from ASA 2 to ASA 3 or ASA 4 to ASA 5 did not impact mortality predictions as expected.

To understand the impact of ASA misclassification, we needed to develop a global estimate of ASA misclassification incidence for the entire NSQIP population, not just the mortalities. As the differential impact between ASAs 2 and 3, and between 4 and 5, was negligible compared to that between ASAs 3 and 4, our study focused on ASA 3 cases only. To objectively estimate the incidence of misclassified ASA 3 patients, a random sample of 50 patients was selected from the 2014 elective NSQIP surgical cases with charted ASA 3 classifications. Additionally, 10 patients of the total 74 emergency cases who were initially charted ASA 3 were randomly selected. These samples were used to estimate the frequency of ASA 3 over- and under-classification. Patient selection was performed by random number generating software (SAS 9.4, Cary, NC).

After correcting the misclassified ASA scores of the random samples, SAS 9.4 was used to simulate the number of expected deaths using the odds ratios for mortality in the published 2014 GVMM and adjusted for the new rates of each ASA class. Both over- and under-classifications were included in the model. The simulation was run 1000 times. As the entered data represented the probabilities of a particular outcome, each run of the simulation generates a number of predicted deaths.

Statistical analyses were performed using JMP 10 and SAS 9.4 (Cary, NC). Contingency analysis using the chi-square test was performed for categorical variables.

## Results

The patient characteristics of our study populations (all cases and mortalities) are shown in Table 2. Patients who died were older and more likely to be outside transfers and emergencies (*p* < 0.05). In addition, certain surgical populations, namely, breast and endocrine surgery patients, had no observed mortality. Characteristics such as gender, race, and Hispanic ethnicity were not different between groups. When evaluating NSQIP variables, the mortality group also had a greater incidence of partially dependent functional status, disseminated cancer, diabetes, hypertension, tobacco use, chronic obstructive pulmonary disease, acute renal failure, dirty wounds, ascites, and ventilator use at the time of surgery (Table 3, *p* < 0.05). A total of 1264 NSQIP essential cases were performed during 2014. Patients transferred from other institutions were more likely to be emergency cases compared with patients who were not transfers (43.5 vs. 7.84%, *p* < 0.05), and transferred patients had a higher proportion of ASAs 3–5 vs. ASAs 1–2 compared with non-transfers (84.38 vs. 49.76%, *p* < 0.05). When comparing our study population to the NSQIP reported total population (*n* = 768,612), our study population had a higher proportion of transferred patients for mortalities (57.6 vs. 29.9%, *p* < 0.05) and survivors (11 vs. 3.9%, *p* < 0.05). Additionally, our study population had a higher number of patients with 3+ risk factors in both mortalities (87.9 vs. 60.1%, *p* < 0.05) and survivors (23 vs. 11.6%, *p* < 0.05) than the NSQIP comparison population.

**Table 2:** Patient Characteristics of the Study Population

Demographics		All cases % (no.)	Mortalities % (no.)	*p* value
Age		56.5 ± 0.47	71 ± 3	< 0.001
Gender	Male	45.3% (573)	57.6% (19)	0.215
	Female	54.7% (691)	42.4% (14)	
Race	White	82.2% (1039)	84.8% (28)	0.896
	Black	10.4% (132)	12.1% (4)	
	American Indian	1.1% (14)	0.0% (0)	
	Asian	1.1% (14)	0.0% (0)	
	Unknown	5.1% (65)	3.0% (1)	
Hispanic ethnicity	3.4% (43)		0.0% (0)	0.624
Emergency		12.1% (153)	70.0% (23)	< 0.001
Transfer		12.1% (153)	60.6% (20)	< 0.001
Surgery type	General surgery	41.0% (518)	51.5% (17)	0.046
	Breast/endocrine	21.5% (272)	0.0% (0)	
	Vascular	18.4% (233)	27.3% (9)	
	Colorectal	10.1% (128)	15.2% (5)	
	Hepatobiliary	5.8% (73)	6.1% (2)	
	Bariatric	3.2% (40)	0.0% (0)

**Table 3:** NSQIP risk factors in the study population

NSQIP variables		All cases % (no.)	Mortalities % (no.)	*p* value
Functional status	Independent	96.2% (1216)	87.9% (29)	0.013
	Partially dependent	3.2% (40)	12.1% (4)	
	Totally dependent	0.6% (8)	0.0% (0)	
Wound class	Clean	55.0% (695)	33.3% (11)	0.003
	Clean/contaminated	27.3% (345)	27.3% (9)	
	Contaminated	7.9% (100)	12.1% (4)	
	Dirty	9.8% (124)	27.3% (9)	
Steroid		4.1% (52)	12.1% (4)	0.050
Ascites		0.5% (6)	9.1% (3)	0.001
Sepsis	SIRS	6.6% (84)	21.2% (7)	< 0.001
	Sepsis	4.7% (59)	18.2% (6)	
	Septic shock	1.0% (13)	24.2% (8)	
Ventilator		1.3% (16)	27.3% (9)	< 0.001
Disseminated cancer		4.2% (53)	24.2% (8)	< 0.001
Diabetes		20.1% (254)	39.4% (13)	0.014
Hypertension		48.7% (616)	75.8% (25)	0.002
CHF		0.6% (8)	3.0% (1)	0.208
Dyspnea	At rest	0.6% (7)	3.0% (1)	0.069
	Moderate exertion	8.4% (106)	15.2% (5)	
	None	91.1% (1151)	81.8% (27)	
Smoker		31.3% (396)	39.4% (13)	0.039
COPD		7.5% (95)	27.3% (9)	< 0.001
Dialysis		2.4% (30)	6.1% (2)	0.194
ARF		0.6% (8)	6.1% (2)	0.025
BMI		30.4 ± 0.24	27.5 ± 1.54	0.054

Our initial medical record review of 33 mortalities showed 18.2% of ASA scores were misclassified, mostly in patients originally scored ASA 3 or ASA 4. 12.1% were under-classified (initially received a lower ASA), and 6.1% were over-classified. Discrepancies between recorded and reclassified ASA scores appeared to be the greatest contributor to the NSQIP predicted mortality (compared to all other factors on the online NSQIP model) particularly when ASA 4 and 5 cases were under-classified as ASA 3. Emergency cases were more likely to have a higher ASA score (*p* < 0.05) and were more likely to be misclassified (*p* < 0.05). Table 4 lists the reclassified ASA scores and the medical rationale for reclassifying the ASA score.

**Table 4:** Reasons for ASA misclassification in the study population

2014 mortalities			
Patient	Charted ASA	Recalculated ASA	Reason for change
1	4	5	Ruptured abdominal aortic aneurysm with intraoperative cardiac arrest
2	3E	5E	Superior mesenteric artery occlusion with bowel ischemia
3	Not recorded	5	Perforated colon with sepsis. Moribund
4	4	3	Reviewer used subsequent cases after complications instead of index case
5	4	3	Several severe systemic comorbidities (poorly controlled diabetes, chronic obstructive pulmonary disease, asthma), but none were a constant threat to life
6	3	4	Active congestive heart failure
2014 all cases sample			
Patient	Charted ASA	Recalculated ASA	Reason for change
1	3	2	Controlled hypertension and asthma, otherwise healthy. Localized Hurthle cell cancer
2	3	4	Stroke within 3 months
3	3	4	Myocardial infarction within 3 months with 14% left ventricular ejection fraction on echocardiogram
4	3E	4E	Perforated small bowel with sepsis
5	3E	2E	Infected thigh hematoma, but not septic. Remote history of supraventricular tachycardia, but otherwise healthy and not on medications
6	3E	4E	Perforated viscus with sepsis
7	3E	4E	Bowel necrosis present on colonoscopy prior to operation
2014 emergency cases sample			
Patient	Charted ASA	Recalculated ASA	Reason for change
1	3E	4E	Perforated viscus
2	3E	5E	Ruptured abdominal aortic aneurysm
3	3E	4E	Perforated diverticulitis
4	3E	4E	Perforated small bowel with sepsis
5	3E	4E	Ongoing crescendo transient ischemic attacks
6	3E	2E	Appendicitis, not septic and no major medical problems

To determine if ASA scores were also systematically misclassified in the all cases population, we reviewed medical records of 50 randomly chosen survivors initially assigned an ASA score of 3. In this random sample, the ASA was misclassified in 16% of patients with five under-classified and two over-classified. A random sample of 10 patients who underwent emergency surgery was also analyzed. Six of these patients had ASA scores that were misclassified (*p* < 0.05) including one over-classification. Table 4 summarizes the factors that led to ASA reclassification.

**Fig. 1:**
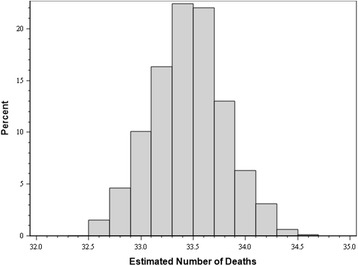
Simulated predicted death adjusting for ASA misclassification. When adjusting for re-classified ASA scores in the sample populations, simulations using the odds ratio of mortality based on the GVMM reports predicted increased mortality matching our institutions observed mortality. Histogram bars depict the percentage of simulations that resulted in the mortality rates shown on the *x*-axis. The number of deaths with the greatest likelihood based on the simulation model was 33.5. The simulation was run 1000 times. Both over-classification and under-classification rates were included in the model

Predicted mortality simulations were then performed using the misclassification rates discovered above. Figure 1 shows the distribution of the results of the simulation model for the ASA 3 misclassifications. Ultimately, the simulation shows the number of predicted deaths was 33.46 (95% CI 33.44–33.48), determined by the median of the curve generated by the histogram. Based upon the simulated estimated deaths, our O/E mortality rate would be 0.9864 (95% CI 0.9858–0.9871), essentially identical to the number of observed deaths in 2014 (33 deaths), whereas the NSQIP report only predicted 30.6 deaths (O/E mortality 1.0784).

## Discussion

Quality improvement initiatives such as the NSQIP and the University Hospital Consortium (UHC) databases are important benchmarking resources, which allow participating hospital systems to assess the quality of care provided at their institutions (Fink et al. 2002). However, when specific quality scoring systems are used to evaluate patient care, it is assumed that the data used to assess patient acuity and outcomes are accurate. The UHC quality benchmarking process utilizes administrative data to “risk-stratify” patient outcomes. Administrative databases require accurate documentation of patient’s medical diagnoses in the medical record to accurately “risk-stratify” patient outcomes. Abstracted databases like NSQIP are assumed to be more accurate in patient risk factor stratification (Steinberg et al. 2008). However, as with any “scoring system,” the users must understand the strengths and weakness of the system to use it properly. NSQIP is designed to predict the risk of complications and mortality based on information about the patients and their medical conditions that is available prior to performing surgery. Consequently, misrepresentation of these variables, such as failure to recognize sepsis or misclassifying the ASA, can significantly underestimate predicted mortality and thereby inaccurately categorize hospitals as being poor performers.

We discovered that at our institution, an academic medical center with many trainees, ASA misclassifications were relatively common (16%), especially in emergencies (60%). Under-classification of ASA scores 4 and 5 as ASA 3 was unexpected. The statistical model we created suggests that with proper ASA classification, our institution’s predicted mortality would have matched our observed mortality. In addition, emergency surgical procedures were most commonly misclassified and transferred patients were most often emergency cases. Thus, the predicted mortality of institutions with a high volume of transferred emergency surgical cases, such as ours, may be artificially reduced from underestimated ASA classifications. This is consistent with data suggesting that emergency cases are prone to high O/E ratios and risk under-classification in general (Hyder et al. 2015).

The reasons why so many ASA scores were misclassified are unclear. As a teaching hospital, it is tempting to assume that junior anesthesia trainees were not as familiar with the ASA score calculation as they should be, or that emergency cases performed on nights and weekends were prone to “erroneous ASA classification” (Gawande et al. 2003). However, Tables 3 and 4 offer some additional insights. First, many patients who were misclassified as ASA 3, when they should have been ASA 4, presented with sepsis or perforated viscus. In reviewing their charts, some of these patients appeared quite well on initial examination; however, their vital signs, laboratory values, and imaging met the Systemic Inflammatory Response Syndrome criteria for sepsis. In addition, all of the ASA 5 patients misclassified as ASA 3 presented with ruptured abdominal aortic aneurysm. On presentation and initial exam, because they were “contained ruptures,” these patients also appeared quite well with minimal abnormalities in their vitals or laboratory values. However, while these patients were clinically well appearing, ruptured aortic aneurysms are by definition granted an ASA 5 on the ASA guidelines, as the natural history for these cases is quick propagation to overt rupture and death. A few under-classified patients had medical histories consistent with ASA 4 as well, such as myocardial infarction within 3 months or ongoing transient ischemic attacks prior to surgery. As such, there appeared to be an over reliance on the subjective physical appearance of the patient at the time of examination, as opposed to factors such as the natural history of their disease process and previous medical history.

The ASA score is a subjective measure of baseline patient illness; however, it is critical that all providers who assign ASAs are doing so in a consistent manner. Our study provides evidence ASA misclassification can significantly impact predicted mortality and supports continued education regarding the potential impact of ASA scoring on O/E mortality in surgical patients. To address this concern, we communicated our findings on ASA misclassification to both the surgery and anesthesia departments and provided education regarding the ASA score and its importance in calculating predicted mortality. We also modified our institutional procedure verification and time out policy by incorporating the ASA score into the surgical time out. Our revised policy requires the attending anesthesiologist to communicate the ASA score to the surgical team as part of the time out. The attending surgeon is required to acknowledge the assigned ASA score prior to starting the operation and initiate a discussion about it if there are concerns about the assigned score. In addition, surgical residents are required to use the online NSQIP calculator for all cases presented in the weekly morbidity and mortality conference. A future study will reevaluate the incidence of misclassified ASAs after such education has been instituted.

There are several limitations to this study. First, it is a single-center retrospective review of NSQIP mortalities in a large academic medical center. Consequently, the results may not apply to participating NSQIP institutions of varying size and type. Future studies being considered include expansion of the current study to multiple institutions, as well as revisiting our ASA misclassification rate after education. Second, the actual model used to calculate NSQIP predicted mortality is proprietary, so we are unable to report exact statistical change in predicted mortality. In the future, NSQIP plans to be increasingly robust. For example, NSQIP models will improve with specific variables collected for complex hepatobiliary cases.

## Conclusion

ASA misclassification significantly impacts observed/expected mortality ratio, and thus, how a particular institution’s safety is viewed. In our review, misclassification, particularly in emergency cases, underestimated the number of predicted deaths by up to 9%. With accurate ASA classification, observed mortality would not have exceeded expected mortality in our institution. Continued education regarding the impact of ASA scoring is essential to ensure accurate O/E mortality data is being used to assess surgical quality at participating NSQIP institutions. Our institution has since instituted a policy that the ASA must be announced during the pre-procedure time out and agreed upon or discussed prior to incision.

## Abbreviations

ASA: American Society of Anesthesia
DNR: Do not resuscitate
GVMM: General Vascular Mortality Model
NSQIP: National Surgical Quality Improvement Project
O/E: Observed to expected
UHC: University Hospital Consortium
